# A FnWRKY17–*FnFLA16* regulatory module controls leaf curling in *Fragaria nilgerrensis*

**DOI:** 10.3389/fpls.2026.1837692

**Published:** 2026-06-23

**Authors:** Xuanrong Zheng, Xiaoqiao Zhu, Huaizheng Wang, Jiangrun Luo, Yunxiong Jiang, Jiajun Lei, Shu Jiang

**Affiliations:** 1 Agronomy and Life Science Department, Zhaotong University, Zhaotong, Yunnan, China; 2 College of Horticulture, Shenyang Agricultural University, Shenyang, Liaoning, China

**Keywords:** cellulose, FnFLA16, FnWRKY17, Fragaria nilgerrensis, leaf curling, strawberry

## Abstract

**Introduction:**

Moderate leaf curling is a desirable trait in crop breeding and has attracted increasing research interest. *A curled leaf* (*cl*) mutant was obtained from a previously developed ethyl methanesulfonate (EMS) mutant library of the strawberry *Fragaria nilgerrensis*; however, the regulatory frameworks and interaction networks responsible for this phenotype remain largely undefined.

**Methods:**

Modifications in cell wall architecture and cellulose content, along with related biological features, were detected in the *cl* mutant. Transcriptomic RNA sequencing was performed to compare gene expression between the *cl* mutant and the wild type (WT). Functional verification was carried out through genetic manipulation. Yeast one- hybrid (Y1H), dual- luciferase (LUC), and β- glucuronidase (GUS) assays were used to test transcriptional regulation. Transient expression assays were also conducted to confirm the role of the identified regulator.

**Results:**

Transcriptomic analysis revealed substantial differential expression of *fasciclin- like arabinogalactan* (*FLA*) genes between the *cl* mutant and WT. Functional verification showed that *FnFLA16* altered cellulose- associated biological properties, thereby promoting leaf curling. Subsequent correlation analyses and quantitative assays identified critical transcriptional regulators. FnWRKY17 was demonstrated to directly bind to the *FnFLA16* promoter, as confirmed by Y1H, LUC, and GUS assays. Transient expression assays further confirmed that FnWRKY17 operates within the FnFLA16- cellulose regulatory framework controlling leaf curling.

**Conclusions:**

These findings reveal a previously unreported FnFLA16- centered working model underlying leaf curling in *F. nilgerrensis*. This study provides new insights into the regulatory mechanisms of leaf curling and identifies potential targets forbreeding programs.

## Introduction

Leaf morphology is a critical agronomic trait that determines optimal plant architecture and regulates photosynthesis, respiration, and transpiration. Moderate leaf curling contributes to a more erect leaf posture, minimizes intra-canopy shading, enhances light interception, and improves photosynthesis (Govaerts et al., 1996; Zhang et al., 2009) and drought tolerance (Lang et al., 2004; Zhang et al., 2009). One of the reasons for the leaf curling is alteration in cell division and elongation, structure, composition, and polarity (Zou et al., 2011), which subsequently modifies the biological properties of the tissue (Liu et al., 2020; Kim et al., 1998; Serrano-Cartagena et al., 1999). For instance, investigations of rice leaf-curling mutants, *Roc5*, *RL14*, and *nrl1*, demonstrated increases in bulliform cell number, size, and cellulose content (Zou et al., 2011; Hu et al., 2010). Similarly, analysis of the *fla16* mutant in *Arabidopsis* showed that the lack of *FLA16* lead to reduced stem length and altered biomechanical characteristics, which are likely associated with decreased cellulose content (Liu et al., 2020). Leaf curling in strawberries has been primarily reported in relation to disease symptoms (Frazier and Posnette, 1957; Rossi et al., 2020; Sababa et al., 2022). However, the underlying mechanisms by which tissue biological properties mediate leaf curling in *F. nilgerrensis* remain unclear.

Arabinogalactan proteins (AGPs) are a diverse group of cell wall glycoproteins that participate in numerous aspects of plant growth and development, including cell division, proliferation, tissue differentiation, stress adaptation, and hormone-mediated signaling (Yang et al., 2007; Lee et al., 2005; Levitin et al., 2008; Coimbra et al., 2009; Park et al., 2003; van Hengel and Roberts, 2003; Liu and Zhang, 2008; Liu et al., 2020; Ma et al., 2018). Fasciclin-like AGPs (FLAs) are distinct subclasses within the AGP superfamily. A defining feature of FLAs is the presence of one or two FAS1 domains, which are thought to facilitate protein-protein interactions (Johnson et al., 2011). These domains are interspersed with regions enriched in glycosylation sites characteristic of AGPs, and many FLAs undergo post-translational modification with a glycosylphosphatidylinositol (GPI) anchor that localizes them to the outer surface of the plasma membrane (Liu et al., 2020). This distinctive structural organization enables FLAs to function as molecular connectors that couple structural reinforcement with signal mediation, thereby influencing the deposition and spatial arrangement of major cell wall polymers, including cellulose and lignin, and consequently altering tissue biomechanical properties. Studies have demonstrated that stem-specific *FLAs* contribute to the maintenance of stem biomechanical properties by regulating cell wall architecture and biosynthesis, with functional roles documented in multiple species, including *Arabidopsis* (Liu et al., 2020), *Oryza sativa* (Jiang et al., 2022a), *Eucalyptus* (Dahiya et al., 2006), *Zinnia* (MacMillan et al., 2015) and *Populus* (Wang et al., 2015, 2017). For instance, in the model plant *Arabidopsis thaliana*, *fla11*, *fla12*, and *fla16* loss-of-function mutants display markedly reduced stem tensile strength and altered elastic modulus, which are closely associated with decreased cellulose accumulation and increased cellulose microfibril angle within the secondary cell wall (Liu et al., 2020; Ma et al., 2022, 2023; MacMillan et al., 2010, 2015). Targeted genetic manipulation of *OreFLA11* and *PtFLA6* expression modifies tissue flexural strength and textile stiffness in *Ostrya rehderiana* and *Populus* through alterations in cell wall composition (Niu et al., 2024; Wang et al., 2015). In addition, FLAs contribute to signal transduction between the intracellular compartment, the extracellular matrix, adjacent tissues, and the external environment, thereby affecting the deposition of cell wall components (MacMillan et al., 2015). The FLA4-FEI signaling pathway exemplifies this dual functionality by regulating the composition and expansion of the root cell wall under saline conditions by linking cell wall organization with stress-responsive signaling (Basu et al., 2016; Griffiths et al., 2016). Accordingly, FLAs are considered to perform both structural and signaling roles through extensive interactions within the extracellular matrix (Liu et al., 2020; Johnson et al., 2003). Overall, the *FLA* gene family plays a central role in establishing and preserving cell wall structure, ensuring the mechanical stability required for plant growth, support, and environmental adaptation. Despite their importance, studies on *FLAs* in strawberry remain limited. The mechanisms by which FLA regulates the biological attributes of curled leaves in strawberry remain insufficiently elucidated, and the upstream transcriptional factor (TF) control of *FLA* genes associated with leaf curling is still poorly understood.

WRKY proteins comprise a family of TFs characterized by a highly conserved WRKYGQK amino acid sequence and a zinc finger-like motif (Rushton et al., 2011). They participate in various tissue growth and development and mediate responses to both biotic and abiotic stresses through sequence-specific binding to DNA promoter regions of target genes (Rushton et al., 2011). Numerous studies have demonstrated that the *WRKY* genes are involved in drought resistance regulation. GhWRKY17, MdWRKY17, MfWRKY7, PoWRKY17, and GhWRKY21 have been implicated in drought tolerance, and their functional mechanisms have been extensively investigated (Yan et al., 2014; Wang et al., 2020; Shan et al., 2021; Huang et al., 2022; Luan et al., 2023). In addition to stress responses, WRKY proteins regulate tissue development processes by modulating cell wall-associated gene expression (Li et al., 2015; Miyamoto et al., 2020). BnWRKY184 simultaneously influences flowering time and secondary wall formation by regulating transcription of cellulose and lignin biosynthetic genes (Yang et al., 2020). *stp* mutants in *Medicago truncatula* and *A. thaliana* exhibiting abnormal secondary wall thickening in pith cells, accompanied by ectopic accumulation of lignin, xylan, and cellulose were isolated, resulting in an approximately 50% increase in stem biomass density in the *STP* (WRKY-TF) mutant (Wang et al., 2010). However, the roles of WRKYs in strawberry tissue development remain largely unexplored.

A *F. nilgerrensis* mutant (*cl*) displaying a stable outward leaf curling phenotype was generated through apical meristem EMS treatment (Jiang et al., 2022b). Comparative analyses of paraffin-embedded sections, agronomic traits, physiological indices, and transcriptomic datasets between mutant *cl* and WT plants were performed to clarify the leaf curling regulatory mechanisms in *F. nilgerrensis*.

## Materials and methods

### Plant materials

An outward leaf curling mutant of *F. nilgerrensis*, designated *cl*, was generated via EMS mutagenesis (Jiang et al., 2022b), with WT plants used as the control. The specific process is as follows. This *cl* mutant was isolated from the plantets of the wild-type *F. nilgerrensis* plant, whose apical meristems were treated with 0.6% ethyl methanesulfonate (EMS) for 6 hours.The *cl* mutant (displaying stable leaf curling) was identified at the seedling stage in M1. In 2024, all plants were cultivated under open-field conditions at the Strawberry Resource Repository of Zhaotong University. Leaf samples were collected and processed following the method of Jiang et al. (2026) and subsequently used for quantitative real-time polymerase chain PCR reaction (qRT-PCR) and gene cloning analyses.

### Quantification of LCI, monosaccharides, and cellulose content, flexural and tensile tests, and histological observation

The leaf length, leaf width, leaf area, leaf thickness and leaf curl index (LCI) was measured using the central leaflet of the third fully expanded leaf in both *cl* mutant and WT plants, following the method described by Jiang et al. (2026). The biomass was measured as the dry weight of the leaves of individual 4-week-old seedlings. They were dried at a constant temperature of 80 °C for 24 to 48 hours until a constant weight was achieved.

For anatomical comparison between *cl* and WT (and CK-treated) leaves, 2-cm segments from both sides of the midrib of mature third leaves were fixed in FAA for 2–4 days, followed by dehydration, clearing, embedding, and staining with toluidine blue (Jiang et al., 2023). Sections were examined using an Olympus BX microscope (Olympus Corporation, Tokyo, Japan).

The monosaccharide content was determined using the TFA (Trifluoroacetic acid) hydrolysis method (Wang et al., 2018). Cellulose was quantified according to the procedure described by Jiang et al. (2024). The flexural and tensile properties were assessed according to Liu et al. (2020), described by MacMillan et al. (2010).

### RNA sequencing and data analysis

Young central leaflets from *cl* mutant and WT were harvested from different plants to form biological repetition. Three biological replicates were generated using independently sourced biological material, with each test consisting of 2 g of tissue. After sampling, the collected leaflets were flash-frozen in liquid nitrogen and stored at −80 °C for transcriptome sequencing. Total RNA was isolated from *cl* and WT samples, and cDNA libraries were constructed and sequenced by LC Corporation (LC Bio, China) following the protocol described by Jiang et al. (2024).

Clean reads were aligned to the *Fragaria vesca* reference genome (https://www.Rosaceae.org/species/fragaria_vesca/genome_v6.0). Principal component analysis (PCA) was performed using TBtools to assess gene expression patterns and biological replicate consistency across samples (Jiang et al., 2024).

Differentially expressed genes (DEGs) between *cl* and WT were identified using a false discovery rate (FDR) threshold of ≤ 0.05 and |log_2_ fold change (FC)| > 1 based on FPKM (Fragments per kilobase of transcripts per million mapped reads) values. TFs exhibiting strong correlations with structural genes (Pearson’s |r| ≥ 0.90) were retrieved from the PlantTFDB database (Jiang et al., 2024). Candidate TFs and structural genes with |log_2_ FC| ≥ 1.5 and FPKM > 20 were visualized using Cytoscape (v3.8.2) and hierarchical clustering heatmaps.

### Total RNA extraction and qRT–PCR analysis

Total RNA was extracted from the *cl* and WT leaves using the CTAB method (Jiang et al., 2024). RNA quality assessment, cDNA synthesis, and qRT–PCR (IQ5 system, Applied Biosystems) were performed as described by Jiang et al. (2024, 2026). The primer sequences are provided in **Supplementary Table 1**.

### Coding sequence, promoter isolation, and sequence alignment

The coding sequences (CDS) of *FnWRKY17* and *FnFLA16* were retrieved from the *F. vesca* version 6.0 (Zhou et al., 2023) transcriptome database and amplified from *F. nilgerrensis* cDNA. The *FnFLA16* promoter fragments were isolated from *F. vesca* v6.0 genomic DNA. Cloning was performed according to the method described by Jiang et al. (2026). To characterize the *FnWRKY17* and *FnFLA16* sequences, multiple sequence alignments were performed using DNAMAN 6.0. The phylogenetic tree was generated using MEGA 7.2 to place them evolutionary relative to other species.

### Genetic transformation

The CDS of *FnFLA16* and *FnWRKY17* were cloned into the pRI101-AN vector with 35S as the promoter to generate pRI101-FnFLA16 and pRI101-FnWRKY17 overexpression constructs. These overexpression constructs were generated via homologous recombination−based one−step cloning (In−Fusion^®^ method). Briefly, the CDS fragments with 15–20 bp vector−homologous extensions were amplified and recombined with linearized pRI101−AN using a recombinant enzyme at 50 °C for 15–30 min. The resulting constructs were verified by Sanger sequencing. The transient RNA interference (RNAi) constructs pTRV2-FnFLA16 and pTRV2-FnWRKY17 were generated for gene down-regulation by inserting gene-specific antisense fragments targeting *FnFLA16* and *FnWRKY17* into the pTRV2 (tobacco rattle virus) vector (Jiang et al., 2024). Transient transformations were performed by infiltrating WT and *cl* plants with pRI101-FnFLA16/pRI101-FnWRKY17 and pTRV2-FnFLA16/pTRV2-FnWRKY17 constructs, respectively. For overexpression: the constructs pRI101-FnFLA16 and pRI101-FnWRKY17 were introduced into *Agrobacterium* strain GV3101. For virus-induced gene silencing (VIGS): pTRV2-FnFLA16 and pTRV2-FnWRKY17 were co-infiltrated with the helper vector pTRV1. Each agrobacterial culture was grown to OD_600_ = 0.8–1.0, resuspended in infiltration buffer (10 mM MES, 10 mM MgCl_2_, 200 μM acetosyringone, pH 5.6), and injected into the abaxial side of fully expanded leaves of 4-week-old potted wild-type (WT) and *cl* mutant plants using a needleless syringe. After infiltration, plants were kept in a growth chamber for 2–3 days before sample collection. Empty vector (pRI101-AN vector or pTRV1-pTRV2) infiltrations were used as controls according to Jiang et al., 2026.

### Yeast one-hybrid

The *FnFLA16* promoter was cloned into the pAbAi vector and integrated into the Y1HGold yeast strain, which was cultured in synthetic defined (SD)/-Ura medium. The *FnWRKY17* and *FnZHD9* encoding sequences were inserted into the pGADT7 vector and transformed into Y1H cells harboring the *FnFLA16* promoter construct for interaction analysis. The detailed procedures were as described by Jiang et al. (2026).

### Dual-luciferase

The effector was the pRI101-FnWRKY17 vector, and the reporter construct was generated by inserting the *FnFLA16* promoter into the pGreenII 0800-LUC vector. The empty LUC vector and pRI101-AN were used as negative controls. All constructs were introduced into *Agrobacterium tumefaciens* GV3101. The confirmed colonies were used to prepare the infiltration suspensions (Jiang et al., 2024). For transient expression analysis, 200 μL of bacterial suspension was infiltrated into the leaves of four-week-old *Nicotiana benthamiana*. The luminescence signals were detected using an *in vivo* imaging system after incubation (Jiang et al., 2024).

### GUS

The effector was the pRI101-FnWRKY17 vector, and the *FnFLA16* promoter was cloned into the pRI201-GUS vector to generate the reporter construct, with pRI101-AN used as the control. All constructs were introduced into *A. tumefaciens* GV3101, and positive colonies were selected to prepare the infiltration suspension. Transient expression assays were performed by agroinfiltration of *N. benthamiana* leaves. The FnWRKY17-FnFLA16 interaction was evaluated by histochemical GUS staining on leaf discs using a GUS staining kit (Biosharp, Anhui, China) (Jiang et al., 2026). GUS activity was measured using the GUS Fluorometric Assay Kit (Coolaber, catalog No. SL7161-50T) following the manufacturer’s instructions. Total protein concentration was determined using the Bradford method (Bio-Rad Protein Assay) to carry out normalization, and relative GUS activity was calculated as the fold change of specific activity in each experimental group relative to the empty vector control.

### Subcellular localization

*FnWRKY17* and *FnFLA16* CDS (without stop codons) were amplified, ligated into vectors, transformed into *Escherichia coli*, and sequenced to generate FnWRKY17-GFP (Green fluorescent protein) and FnFLA16-GFP fusion constructs. The pCAMBIA1300-based vector carrying a GFP reporter gene was used for nuclear localization analysis, together with a nuclear marker (DAPI staining). Whereas pY-GFP-NAA60 was used for plasma membrane localization analysis. The localization markers included NAA60-mkate2 (plasma membrane) and DAPI (nucleus). The *Agrobacterium* strain GV3101 was cotransformed with recombinant plasmids labeled with mkate fluorescence, followed by infiltration into the protoplast of *N. benthamiana*. The fluorescence of green fluorescent protein (GFP) was observed using a confocal laser scanning microscope (TCS SP8; Leica, Wetzlar, Germany) following the method described by Ji et al. (2022) and Linster et al. (2020). The pCAMBIA1300-GFP and pY-GFP-NAA60 constructs were used as controls.

### Statistical analyses

Statistical analyses were performed using the SPSS software (v19). Data are presented as mean ± standard deviation (SD) and were obtained from at least three independent biological replicates, each comprising three technical replicates. To ensure that the three biological replicates were truly independent, we used independently sourced biological material for each replicate; Performed all steps from sampling to measurement separately for each replicate. For each biological replicate, we first averaged its three technical replicates to obtain a single representative value. Then, we used the three biological replicate values (n = 3) for statistical analysis. Each independent experiment was performed on independently prepared samples and was considered one biological replicate. The degrees of freedom for all t-tests were calculated as number of replicates−1, consistent with standard biostatistical practice (Sokal and Rohlf, 1970). Statistical significance was assessed using Student’s t-test, with a threshold of *P* ≤ 0.05.

## Results

### LCI index and histological observations of the *cl* mutant in *F. nilgerrensis*

**Figure 1:**
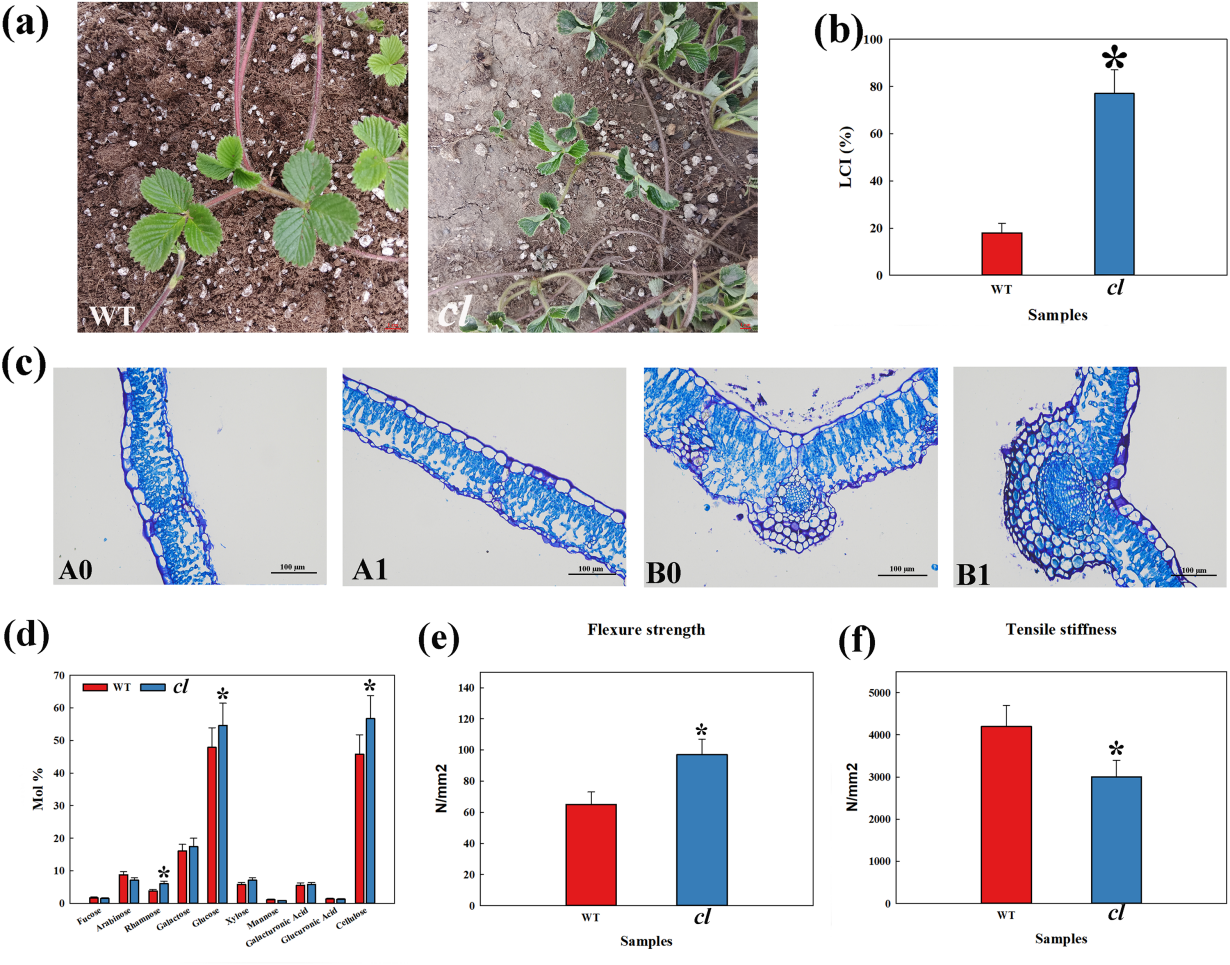
Phenotypic characterization, physiological indexes and paraffin section of *cl* mutant and WT in *F. nilgerrensis*. **(a)** WT: wild type plant. *cl*: curled leaf mutant. (bar =1 cm) **(b)** LCI value of leaves. **(c)** Paraffin section of leaves. (A0, A1). Upper and lower epidermis of leaf (bar =100 μm). (B0, B1) Vascular bundle of leaf (bar =100 μm). 1 referred to *cl* mutant, and 0 referred to WT. **(d)** The content of monosaccharides and cellulose in leaves. **(e, f)** Biomechanical tests on leaf flexure strength and tensile stiffness. Bars indicate SD of means and ‘*’ indicate significant differences among treatments according to Student’s t test (*P* < 0.05).

The *cl* mutant displayed a stable phenotype characterized by persistent inward leaf curling across four consecutive annual growth seasons (**Figure 1a**). The statistical results of leaf traits indicated that there were significant differences in each trait between the WT and *cl*. Compared with the WT, the leaf length, leaf width, leaf area and biomass of the *cl* mutant were significantly reduced, and leaf color became darker (**Table 1**). In particular, the LCI was calculated to quantify the morphological differences between the *cl* and WT plants. The *cl* mutant showed a substantially elevated LCI (78%), corresponding to an 88.97% increase relative to the WT (**Table 1**; **Figure 1b**).

**Table 1:** Trait statistics of WT and *cl* leaves.

Traits Samples	Shape	Color	LCI (%)	Length (cm)	Width (cm)	Area (cm2)	Thickness (mm)	Biomass(g/seedling)
WT	Ellipse	Green	8.7 ± 0.10	3.23 ± 0.33	2.87 ± 0.29	9.27 ± 0.95	0.30 ± 0.04	1.17 ± 0.17
cl	Curling	Dark green	78 ± 8.5 *	2.31 ± 0.23 *	1.17 ± 0.17 *	2.77 ± 0.29 *	0.34 ± 0.05	0.67 ± 0.07 *

‘*’ indicates significant differences between *cl* and WT according to Student’s t-test (*P* < 0.05).

Transverse fresh leaf sections were prepared by paraffin embedding, stained with toluidine blue to visualize cell wall structures, and examined to assess potential morphological differences between the cell walls of the *cl* and WT (**Figure 1c**). The results revealed pronounced structural alterations in the vascular bundles and upper epidermis of *cl* leaves compared with those of WT leaves (**Figure 1c**). Quantitative analysis of transverse sections showed that the vascular bundle area was significantly increased in *cl* leaves compared with that in WT leaves (**Table 2**; **Figure 1c**), whereas the upper epidermal cell size was significantly reduced in *cl* leaves. The average number of upper epidermal cells and vascular bundles cells was determined from five transverse sections (**Table 2**; **Figure 1c**), and the total cell count observed in the leaf indicates that *cl* contained significantly more cells than WT (**Table 2**; **Figure 1c**).

**Table 2:** Tissue and cellular morphology analysis of WT and *cl* leaves.

Samples Parameter	*cl* (Mean ± SD)	WT (Mean ± SD)
Upper epidermal cell size (µm2)	109.53 ± 10.77 *	143.75 ± 15.79
Number of upper epidermal cells	23.33 ± 2.98 *	18.0 ± 2.57
Number of vascular bundle cells	115.73 ± 10.75 *	55.63 ± 6.25
Vascular bundle area (mm2)	0.64 ± 0.04 *	0.25 ± 0.07

‘*’ indicates significant differences between *cl* and WT according to Student’s t-test (*P* < 0.05).

The monosaccharide composition of leaf cell walls was analyzed using the TFA method to determine whether cell wall compositional changes occur in *cl* mutant leaves. The results revealed clear differences in several monosaccharides between *cl* and WT (**Figure 1d**). The contents of rhamnose (Rha), galactose (Gal), glucose (Glc), xylose (Xyl), and galacturonic acid (Gal-UA) were increased in *cl* leaves compared with the WT, whereas the contents of fucose (Fuc), arabinose (Ara), mannose (Man), and glucuronic acid (Glc-UA) were reduced (**Figure 1d**). Notably, the Rha and Glc levels were significantly higher in *cl* than in WT (**Figure 1d**). Previous studies have indicated that Rha and Glc are characteristic monosaccharides of RG-I (Rhamnogalacturonan I) and cellulose, respectively, suggesting that these polysaccharides were altered in the *cl* mutant. Cellulose, a major structural component of the cell wall, was further quantified in the *cl* and WT samples, revealing an increase of approximately 15.9% in *cl* leaves (**Figure 1d**).

Cell wall composition alterations influence the biomechanical properties of tissues. Therefore, leaf biomechanical assays, including flexural strength and tensile stiffness measurements, were conducted. Flexural tests showed that *cl* leaves exhibited significantly greater strength than WT leaves (**Figure 1e**), whereas tensile strength tests indicated a significant reduction in *cl* stiffness (**Figure 1f**).

### Quality assessment of transcriptome data and identification of DEGs in *cl* mutant of *F. nilgerrensis*

**Figure 2:**
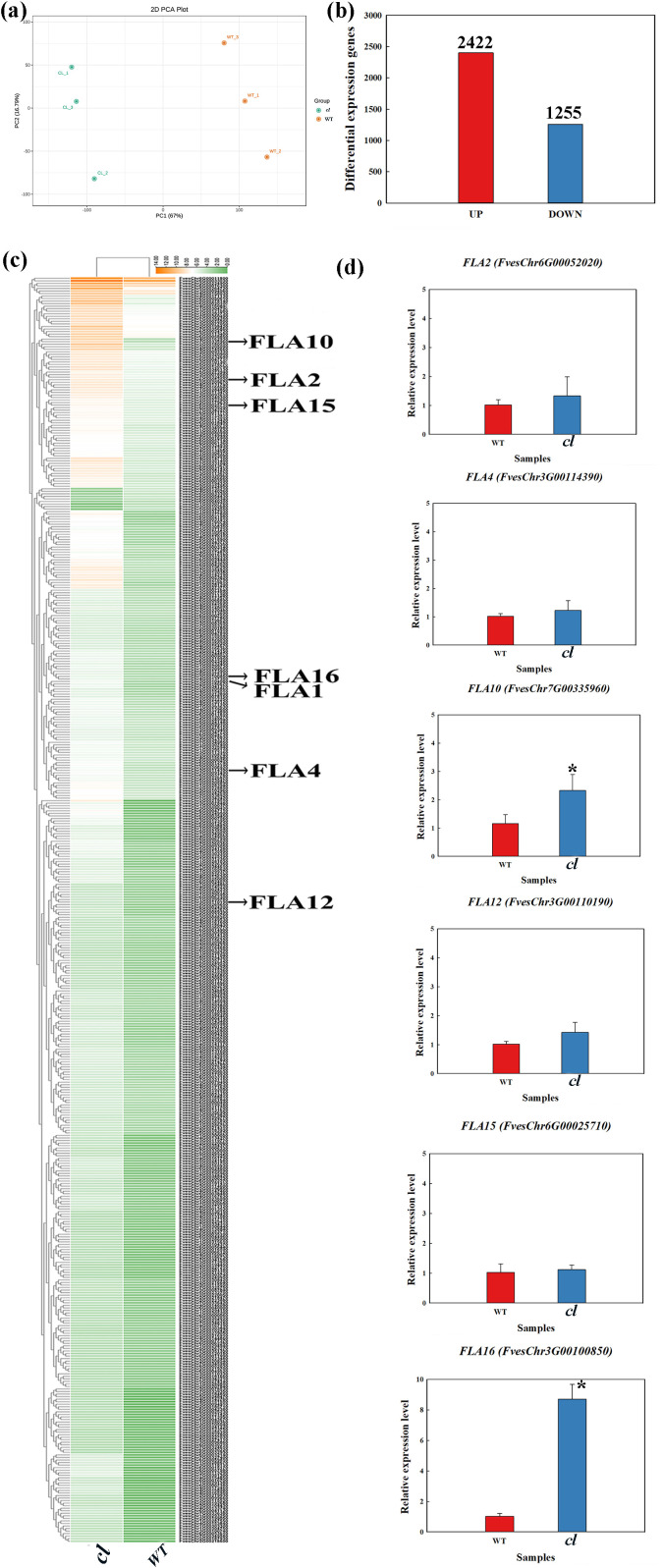
Quality control analysis and overview of differential DEGs of two samples. **(a)** Score plot of principal components analysis based on mass spectrometry data. **(b)** Pairwise comparison of two samples. Red bar represents differential up-regulated genes, and blue bar represent differential down-regulated genes. **(c)** Clustering Heatmap of 499 differential genes. **(d)** Expression level of *FLAs*.

To identify DEGs (Differentially expressed genes) associated with leaf curling, transcriptome sequencing was performed, namely *cl1*, *cl2*, *cl3*, WT1, WT2, and WT3. The raw reads ranged from 34.11 to 43.44 M across the samples. After quality filtering, each sample yielded a value of 5.00–6.37 Gb of clean data, with 75.66–75.86% of reads mapping to the *F. vesca* v6.0 reference genome. PCA revealed strong correlations among the three biological replicates (**Figure 2a**; **Supplementary Table 2**).

DEGs between *cl* and WT were identified using thresholds of *P* < 0.05 and |log_2_ fold change (FC)| >1, resulting in 3, 677 DEGs, including 2, 422 upregulated and 1, 255 downregulated genes (**Figure 2b**; **Supplementary Table 3**). To further refine candidate genes potentially associated with leaf-curling, more stringent criteria (*P* < 0.05, |log_2_FC| > 1.5, FPKM > 10) were applied, yielding 499 DEGs (**Figure 2c**; **Supplementary Table 3**). Among these, several *FLA* family genes, including *FLA16* (FvesChr3G00100850), *FLA15* (FvesChr6G00025710), *FLA2* (FvesChr6G00052020), *FLA10* (FvesChr7G00335960), *FLA12* (FvesChr3G00110190), *FLA1* (FvesChr3G00104080), and *FLA4* (FvesChr3G00114390), showed elevated expression in the *cl* mutant compared with WT plants, suggesting their potential involvement in leaf curling (**Supplementary Table 3**). To validate the RNA-seq results, qRT–PCR analysis was performed on six *FLA* genes in *cl* and WT samples. The qRT–PCR data were consistent with the transcriptome sequencing results, confirming that *FnFLA16* was significantly upregulated in the *cl* mutant than in the WT plants (**Figure 2d**).

### Identification, cloning of the coding sequence, and subcellular localization of *FnFLA16* in the *cl* mutant of *F. nilgerrensis*

**Figure 3:**
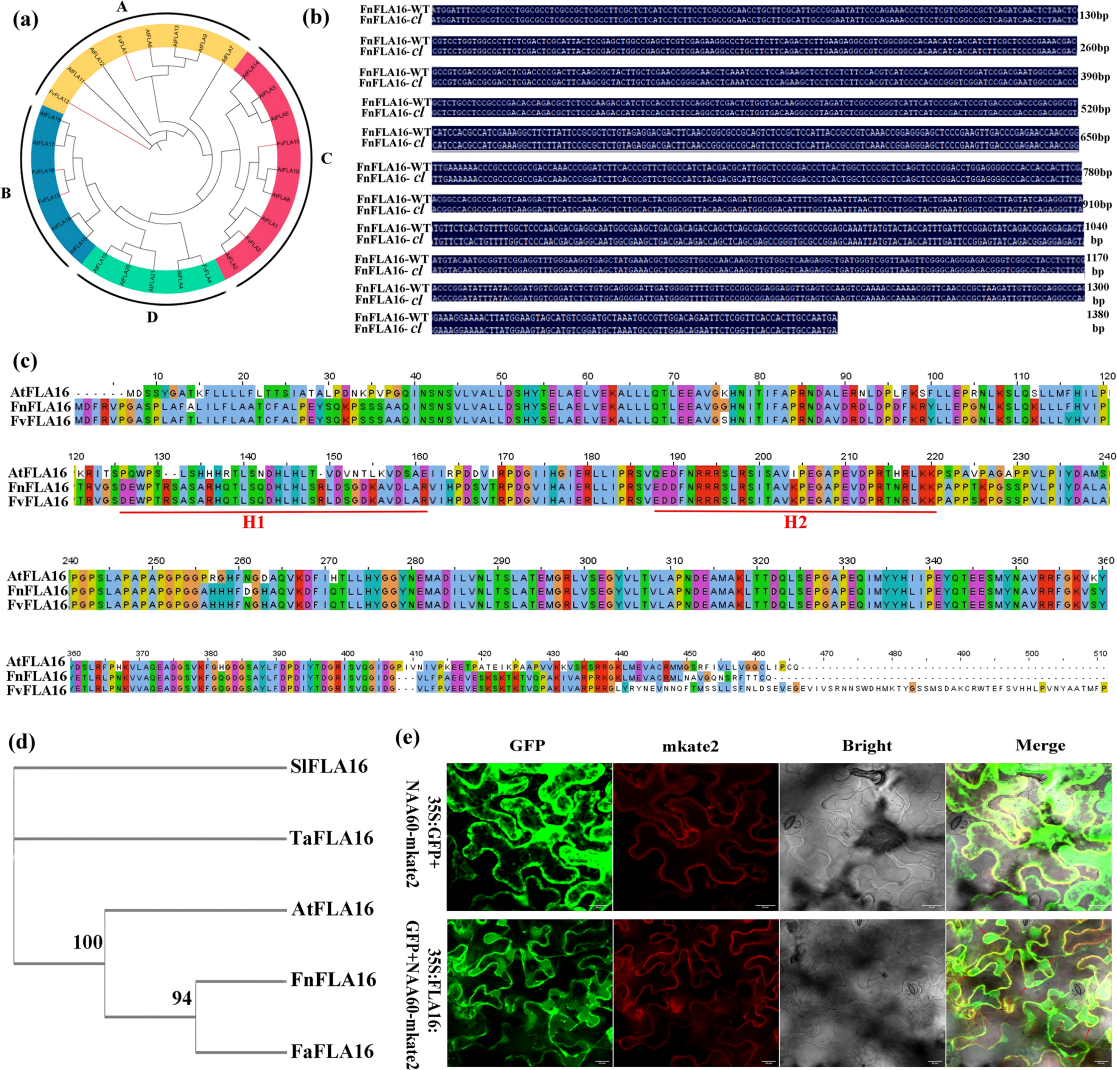
Identification and analysis of FnFLA16. **(a)** Phylogeny of FLAs from *A. thaliana* and *F. nilgerrensis*. **(b)** CDS sequences of *FnFLA16* in *cl* and WT samples. **(c)** Amino acid sequences alignment and comparison of FnFLA16, AtFLA16 and FvFLA16. Identical residues are highlighted on a colour background, and similar residues are highlighted on a white background. **(d)** Phylogeny of FLAs from different crops involved in the regulation of plant resistance and development. **(e)** Subcellular localization. The strawberry gene sequences from the *F. vesca* genome ver6.0 can be downloaded from the Genome Database for Rosaceae (https://www.rosaceae.org) with accession numbers: *FvFLA16*, FvesChr3G00100850; Protein sequences used in the phylogenetic tree can be downloaded from NCBI and Tair with accession numbers: *SlFLA16*, XP_004243392.1; *TaFLA16*, ABI95406.1; *AtFLA1*, At5g55730; *AtFLA2*, At4g12730; *AtFLA3*, At2g24450; *AtFLA4*, At3g46550; *AtFLA5*, At4g31370; *AtFLA6*, At2g20520; *AtFLA7*, At2g04780; *AtFLA8*, At2g45470; *AtFLA9*, At1g03870; *AtFLA10*, At3g60900; *AtFLA11*, At5g03170; *AtFLA12*, At5g60490; *AtFLA13*, At5g44130; *AtFLA14*, At3g12660; *AtFLA15*, At3g52370; *AtFLA16*, At2g35860; *AtFLA17*, At5g06390; *AtFLA18*, At3g11700; *AtFLA19*, At1g15190; *AtFLA*20, At5g40940; *AtFLA21*, At5g06920.

Given the potential role of *FLA* genes in leaf development, a phylogenetic analysis was performed using seven identified *FLA* genes from *Arabidopsis* along with *FLA* family members. Clustering of *FvFLA1*, *FvFLA2*, and *FvFLA10* with their *Arabidopsis* counterparts (*AtFLA6*, *AtFLA2*, and *AtFLA10)* was observed. Similarly, *FvFLA12* and *FvFLA4* showed close relationships with *AtFLA12* and *AtFLA4*, both of which are implicated in stress signaling pathways. *FvFLA16* and *FvFLA15* exhibited a strong correlation with *AtFLA16* and *AtFLA15*, which have been associated with plant growth and development (**Figure 3a**). *FnFLA16* was selected as a candidate gene for further investigation based on expression patterns and phylogenetic relationships.

To further examine *FnFLA16*, its CDS was amplified from the leaf tissues of both *cl* and WT plants. The *FnFLA16* CDS was 1, 380 bp long and encoded a polypeptide of 460 amino acids (**Figure 3b**). Alignment of the deduced amino acid sequence confirmed the presence of conserved H1 and H2 DNA-binding domains in FnFLA16 (**Figure 3c**), thereby confirming its assignment to this gene family. Phylogenetic assessment using representative FLA16 homologs from diverse plant species showed that the FnFLA16 protein clustered closely with AtFLA16, indicating probable functional similarity (**Figure 3d**). Subcellular localization assays demonstrated that the *FnFLA16*-encoded protein was predominantly distributed within the periplasmic region between the plasma membrane and the cell wall (**Figure 3e**).

### Role of *FnFLA16* in regulating leaf curling in *cl* mutant of *F. nilgerrensis*

Despite the compact structure of strawberry leaves, microscopic observation and qRT-PCR confirmed that infiltrated pRI101-FnFLA16 solution spread via vascular bundles to secondary and tertiary veins. Transgene expression in leaf tips 2–3 cm from the infiltration site reached 15–20% of that in the infiltrated zone (**Supplementary Figure 1**). This aligns with previous studies (Meng et al., 2009; Hael-Conrad et al., 2020; Juranić et al., 2020; Jang et al., 2024; Thagun and Kodama, 2025), indicating that local agroinfiltration can drive sufficient distal transgene expression to induce observable phenotypic changes.

**Figure 4:**
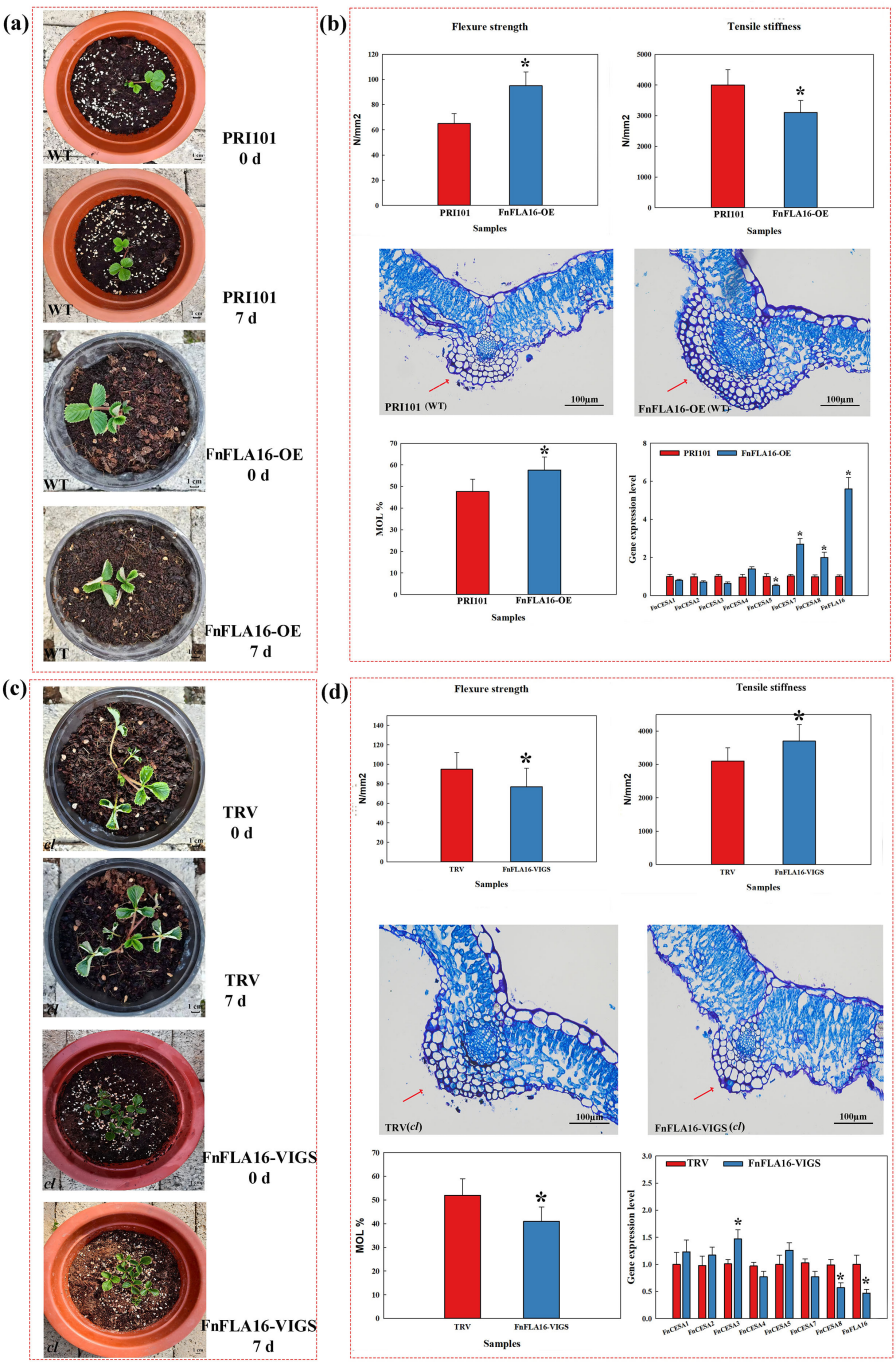
FnFLA16 is correlated with leaf curling in the *cl* mutant of *F. nilgerrensis*. **(a)** Phenotype observation after 7 days of *Agrobacterium*-mediated transient transformation. Above: WT plants infiltrated with empty vector pRI101-empty (negative control); below: WT plants overexpressing *FnFLA16* (FnFLA16-OE). **(b)** Biomechanical and histological analyses of leaves from pRI101-empty and FnFLA16-OE plants (n = 3 biological replicates per group, each replicate consisting of 3 technical measurements). Upper panel: flexure strength (left) and tensile stiffness (right). Middle panel: toluidine blue staining of transverse leaf sections showing cell wall morphology; arrows indicated the increased area of vascular bundles and the increased number of vascular bundle cells in FnFLA16-OE transiently transformed plants. Scale bar = 100 μm. Lower panel left: cellulose content. Lower panel right: relative expression levels of cellulose synthase-related genes (*FnCESA1*, *FnCESA2*, *FnCESA3*, *FnCESA4*, *FnCESA5*, *FnCESA7*, *FnCESA8* and *FnFLA16*) measured by qRT-PCR. **(c)** Phenotype observation at 7 days post-infiltration with TRV-based virus-induced gene silencing (VIGS). Above: *cl* plants infiltrated with empty vector control (TRV1-TRV2) (negative control); below: *cl* plants infiltrated with TRV1-TRV2-*FnFLA16* (FnFLA16-VIGS). **(d)** Biomechanical and histological analyses of leaves from TRV1-TRV2 and FnFLA16-VIGS plants. Upper panel: flexure strength (left) and tensile stiffness (right). Middle panel: toluidine blue staining of transverse leaf sections showing cell wall morphology; arrows indicated the decreased area of vascular bundles and the decreased number of vascular bundle cells in VIGS transiently transformed plants. Scale bar = 100 μm. Lower panel left: cellulose content. Lower panel right: relative expression levels of cellulose synthase-related genes (*FnCESA1*, *FnCESA2*, *FnCESA3*, *FnCESA4*, *FnCESA5*, *FnCESA7*, *FnCESA8* and *FnFLA16*) measured by qRT-PCR. Data were presented as mean ± SD. ‘*’ indicated significant differences among treatments according to Student’s t test (*P* < 0.05).

Previous studies have indicated that members of the FLA family regulate the biological properties of plant tissues. Given that the *cl* mutant had markedly altered polysaccharide content, flexural strength, and tensile stiffness, the functional role of *FnFLA16* in the *cl* mutant was investigated. Transient transformation of WT strawberry leaves was performed using pRI101-empty (CK) and pRI101-FnFLA16 vectors. Phenotypes and related indices were assessed 7 days after infiltration. The leaves of the FnFLA16-OE transiently transformed plants exhibited pronounced curling (**Figure 4a**).

Flexural tests showed significantly greater strength in FnFLA16-OE leaves than in CK (**Figure 4b**), whereas tensile tests indicated a marked reduction in stiffness. Toluidine blue staining of transverse sections revealed that vascular bundle area and vascular bundle cell number were significantly increased in FnFLA16-OE transiently transformed plants relative to CK (**Figure 4b**). The cellulose content in FnFLA16-OE leaves was also significantly higher than that in CK (**Figure 4b**). To determine whether cellulose biosynthesis-related genes were affected, transcript levels of primary wall *FnCESA* genes (*FnCESA1*, *FnCESA2*, *FnCESA3*, and *FnCESA5*) and secondary wall *FnCESA* genes (*FnCESA4*, *FnCESA7*, *and FnCESA8*) in FnFLA16-OE were quantified by qRT-PCR. No significant differences in transcript levels were observed for *FnCESA1*, *FnCESA2*, and *FnCESA3* between FnFLA16-OE and CK leaves (**Figure 4b**), whereas *FnCESA5* was significantly reduced. Conversely, *FnCESA7* and *FnCESA8* transcripts were markedly elevated in FnFLA16-OE leaves, whereas *FnCESA4* showed no significant change (**Figure 4b**).

*FnFLA16* was transiently silenced in the *cl* mutant using the TRV1-TRV2-FnFLA16 construct, with TRV1-TRV2 serving as CK. Phenotypes of the *FnFLA16-*silenced *cl* and TRV1-TRV2 transiently transformed plants were observed. The degree of leaf curling was visibly reduced in *FnFLA16*-silenced transiently transformed plants compared with TRV1-TRV2 transiently transformed plants (**Figure 4c**). The biomechanical indices showed trends opposite to those observed in FnFLA16-OE transiently transgenic plants (**Figure 4d**). The toluidine blue staining test demonstrated that the vascular bundle area and cell number were decreased in *FnFLA16-*silenced transiently transformed plants compared with CK (**Figure 4d**). The cellulose content was also reduced. Gene expression analysis revealed patterns that were opposite to those observed in FLA16-OE leaves (**Figure 4d**). Collectively, these results demonstrate that FnFLA16 modulates cell wall components and associated biological characteristics of leaves in *F. nilgerrensis cl* mutant, thereby contributing to leaf curling.

### Identification of upstream transcription factors regulating *FnFLA16*

**Figure 5:**
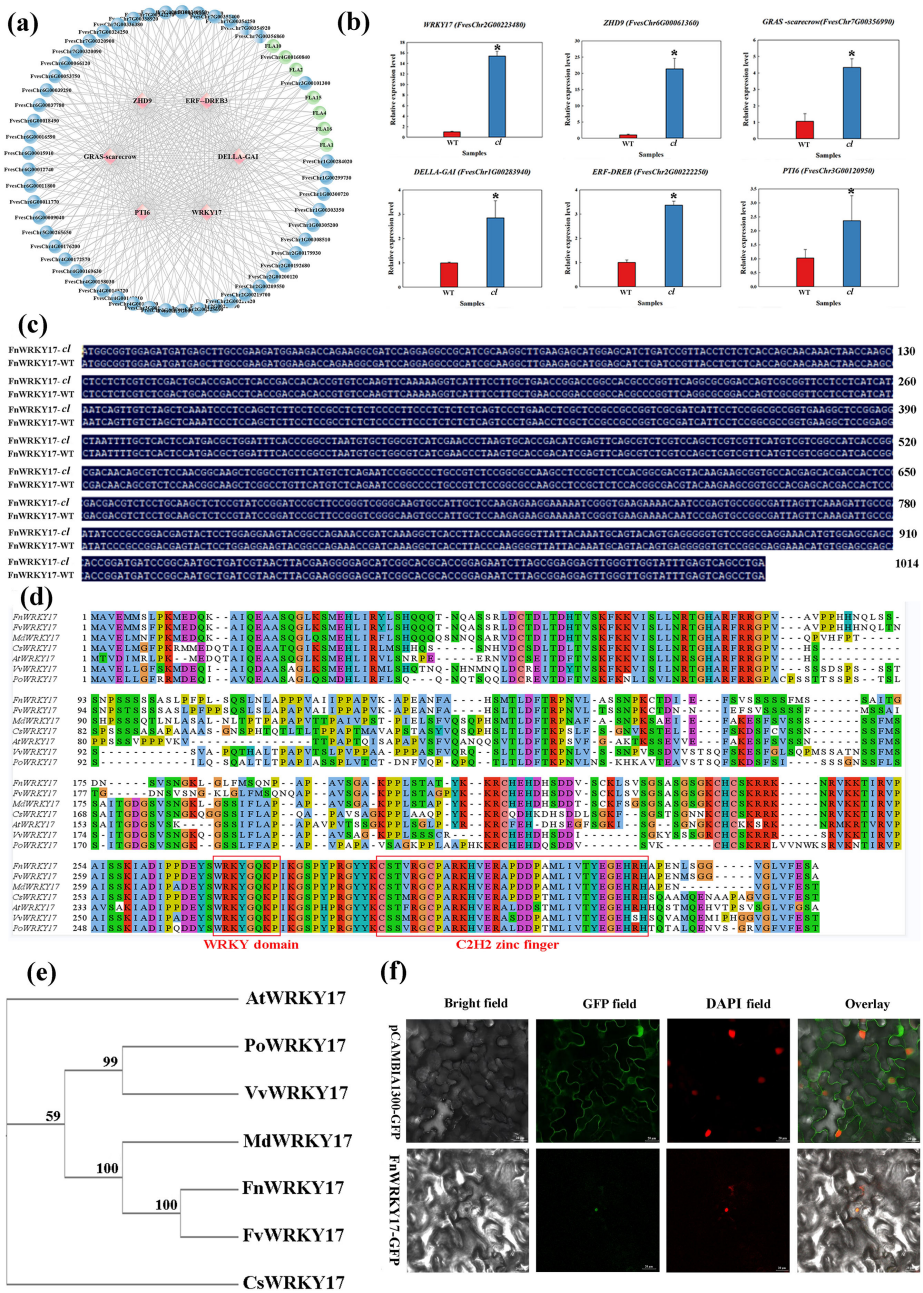
Identification and analysis of FnWRKY17. **(a)** Correlation network between 58 DEGs and 6 TFs. **(b)** Expression levels of different TFs. **(c)** CDS sequences of FnWRKY17 in *cl* and WT samples. **(d)** Amino acid sequences alignment and comparison of FnWRKY17, FvWRKY17, MdWRKY17, CsWRKY17, AtWRKY17, VvAtWRKY17 and PoWRKY17. Identical residues are highlighted on a colour background, and similar residues are highlighted on a white background. **(e)** Phylogeny of WRKY17 from different crops involved in the regulation of plant resistance and development. **(f)** Subcellular localization. Scare bar: 20 µm. The strawberry gene sequences from the *F. vesca* genome ver6.0 can be downloaded from the Genome Database for Rosaceae (https://www.rosaceae.org) with accession numbers. Protein sequences used in the phylogenetic tree can be downloaded from NCBI with accession numbers: *FvWRKY17*, FvesChr2G00223480.1; *AtWRKY17*, AT1G75240; *CsWRKY17*, XP_006483548.1; *VvWRKY17*, XP_002262775.1; *PoWRKY17*, WRI02165.1; *MdWRKY17*, Md12G1181000.

Correlation analysis between six *FLA* genes (*FLA16*, *FLA15*, *FLA2*, *FLA1, FLA10* and *FLA4*) and additional 493 DEGs was performed to elucidate the regulatory basis of *FLAs*-mediated leaf curling (**Supplementary Table 3**) (|r| ≥ 0.90, |log_2_ FC| ≥ 1.5, *P* ≤ 0.05). This approach identified 64 key DEGs, including six differentially expressed TFs: *DELLA-GAI* (FvesChr1G00283940), *GRAS-scarecrow* (FvesChr7G00356990), *PTI6* (FvesChr3G00120950), *ERF-DREB3* (FvesChr2G00222250), *WRKY17* (FvesChr2G00223480), and *ZHD9* (FvesChr6G00061360) (FPKM > 20). A co-expression network constructed in Cytoscape among these 6 TFs and additional 58 DEGs revealed that *WRKY17* displayed the strongest correlation with *FLA16* and *FLA2*, whereas *ZHD9* correlated closely with *FLA15* (**Figure 5a**; **Supplementary Table 4**). DELLA-GAI was strongly correlated with *FLA10* and *FLA4*, whereas PTI6 was associated with *FLA1* (**Supplementary Table 3**). qRT–PCR validation of six TFs confirmed significantly elevated *FnWRKY17* and *FnZHD9* expression in *cl* mutants compared with WT plants (**Figure 5b**), indicating the central roles of *FnWRKY17* and *FnZHD9* in the *cl* regulatory network.

To characterize *FnWRKY17*, its CDS was amplified from the leaves of both *cl* mutant and WT plants. The *FnWRKY17* CDS measured 1, 014 bp and encoded a 337-amino acid protein in both the *cl* and WT samples (**Figure 5c**). An overexpression vector for *FnWRKY17* was subsequently generated. The FnWRKY17 protein contained a WRKY domain and a C2H2-type zinc finger at the C-terminus, confirming its identity as a WRKY family member (**Figure 5d**). Phylogenetic comparison with representative WRKY17 proteins from diverse plant species showed close clustering with MdWRKY17, indicating probable functional conservation (**Figure 5e**). Subcellular localization assays further demonstrated the exclusive nuclear accumulation of the FnWRKY17-GFP fusion protein, whereas the control pCAMBIA1300::GFP signal was distributed throughout the cell, supporting the role of FnWRKY17 as a nuclear-localized TF with transcriptional activity (**Figure 5f**). Jiang et al. (2026) previously showed that the *FnZHD9* CDS is 1083 bp long; phylogenetic and sequence alignment analyses assigned it to the ZHD family, and it functions as a transcription factor localized to the nucleus.

**Figure 6:**
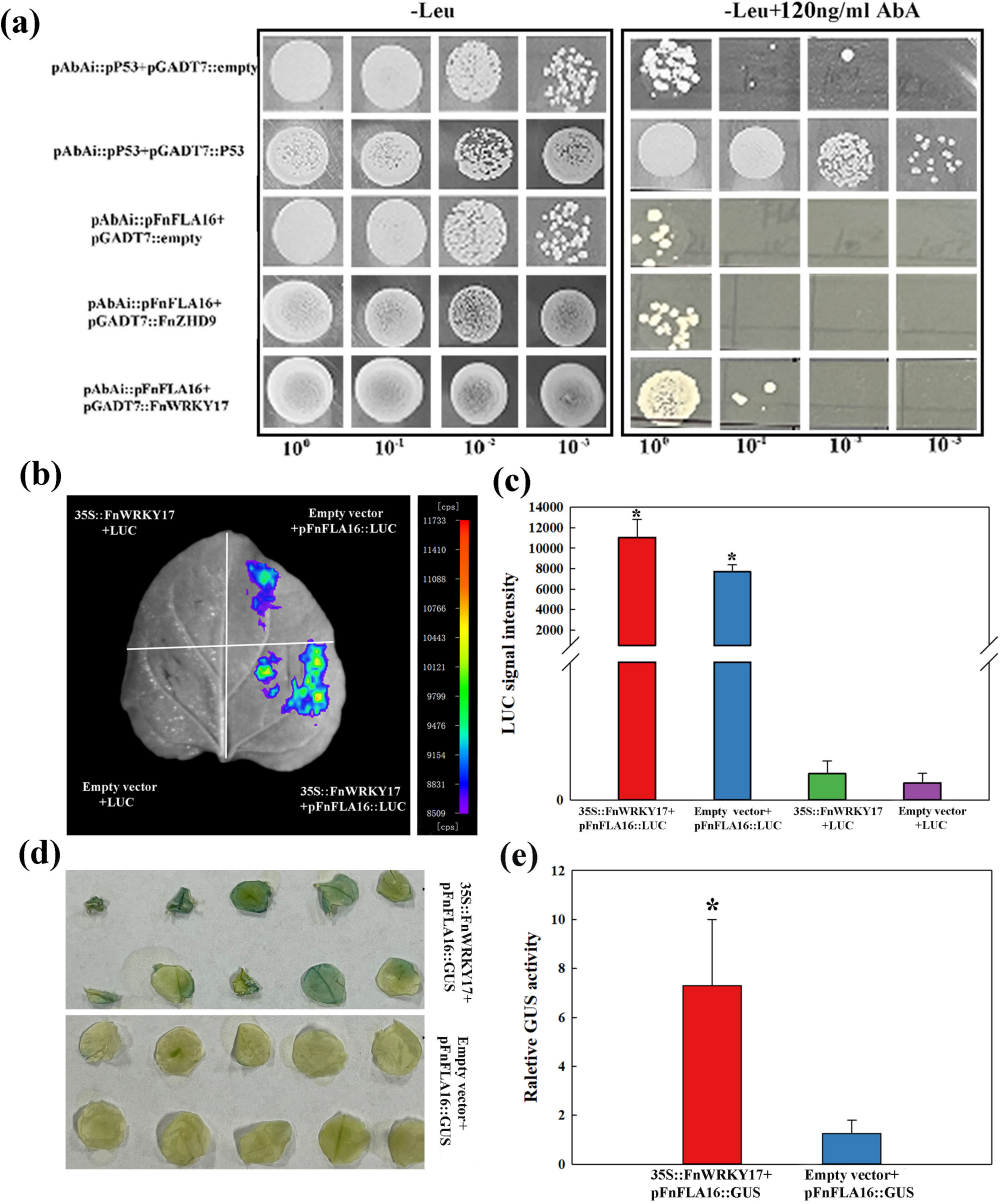
FnWRKY17 positively regulates the target gene *FnFLA16* in the *cl* mutant of *F. nilgerrensis*. **(a)** The interaction between FnWRKY17/FnZHD9 and *FnFLA16* was verified by Yeast one hybrid (Y1H) analysis, using pGADT7::FnWRKY17 or pGADT7::FnZHD9 as the prey, pAbAi::pFnFLA16 as the bait. pAbAi::pP53+pGADT7::empty, pAbAi::pP53+pGADT7::P53, pAbAi::pFnFLA16+pGADT7::empty were respectively the negative and positive controls. **(b)** Dual-luciferase **(LUC)** assays in the *N. benthamiana* leaves transiently expressing the effector (35S::FnWRKY17) with reporter containing proFnFLA16 (pFnFLA16::LUC). 35S::FnWRKY17+LUC, empty (35S)+pFnFLA16::LUC, empty+LUC were respectively the negative and positive controls. **(c)** LUC signal intensity. **(d)** GUS assays in the *N. benthamiana* leaves transiently expressing the effector (35S::FnWRKY17) with reporter containing proFnFLA16 (pFnFLA16::GUS). Empty (35S)+pFnFLA16::GUS was the control. **(e)** Relative GUS activity. Bars indicate SD of means, and ‘*’ indicate significant differences among treatments according to Student’s t test (*P* < 0.05).

Given the pivotal role of FnFLA16 inferred from expression and functional analyses, Y1H assays were performed to evaluate promoter binding by *FnWRKY17* and *FnZHD9*. The pAbAi::pFnFLA16 construct was introduced into yeast cells and cultured on Ura-deficient solid medium for 3–5 days. The pGADT7::FnWRKY17 and pGADT7::FnZHD9 constructs were then transformed into yeast strains carrying pAbAi::pFnFLA16 and grown on Leu-deficient solid medium for 3–5 days. Point-to-point assays on Leu-deficient medium supplemented with AbA indicated that only FnWRKY17 bound to the *FnFLA16* promoter *in vitro* (**Figure 6a**), leading to the selection of FnWRKY17 for subsequent functional analyses.

A LUC assay was performed to determine whether FnWRKY17 modulates the transcriptional activity of the *FnFLA16* promoter. The *FnFLA16* promoter was ligated upstream of the LUC reporter to generate the pFnFLA16::LUC promoter construct, whereas the 35S promoter drove the *FnWRKY17* CDS to create 35S::FnWRKY17. These constructs were co-introduced into tobacco leaves via *Agrobacterium*-mediated transient expression, and luminescence signals were recorded using an *in vivo* fluorescence imaging system. The empty and LUC vectors were used as controls. Co-expression of pFnFLA16::LUC with 35S::FnWRKY17 markedly enhanced *FnFLA16* promoter activity compared with controls (**Figure 6b**), as evidenced by significantly increased LUC signal intensity (**Figure 6c**).

Subsequently, a GUS reporter assay was performed to determine whether *FnFLA16* functions downstream of FnWRKY17. The *FnFLA16* promoter was ligated upstream of the GUS reporter to generate pFnFLA16::GUS, and 35S::FnWRKY17 was used as the effector construct. Following *Agrobacterium*-mediated transient expression in tobacco leaves, leaf discs were stained with histochemical GUS. The pRI101 empty vector was used as the control. Leaf discs coexpressing pFnFLA16::GUS with 35S::FnWRKY17 produced blue staining relative to the control (**Figure 6d**) and significantly elevated GUS activity (**Figure 6e**).

These findings demonstrate that FnWRKY17 directly binds to the *FnFLA16* promoter and functions as a transcriptional regulator.

### Role of *FnWRKY17* in regulating leaf curling in *cl* mutant of *F. nilgerrensis*

**Figure 7:**
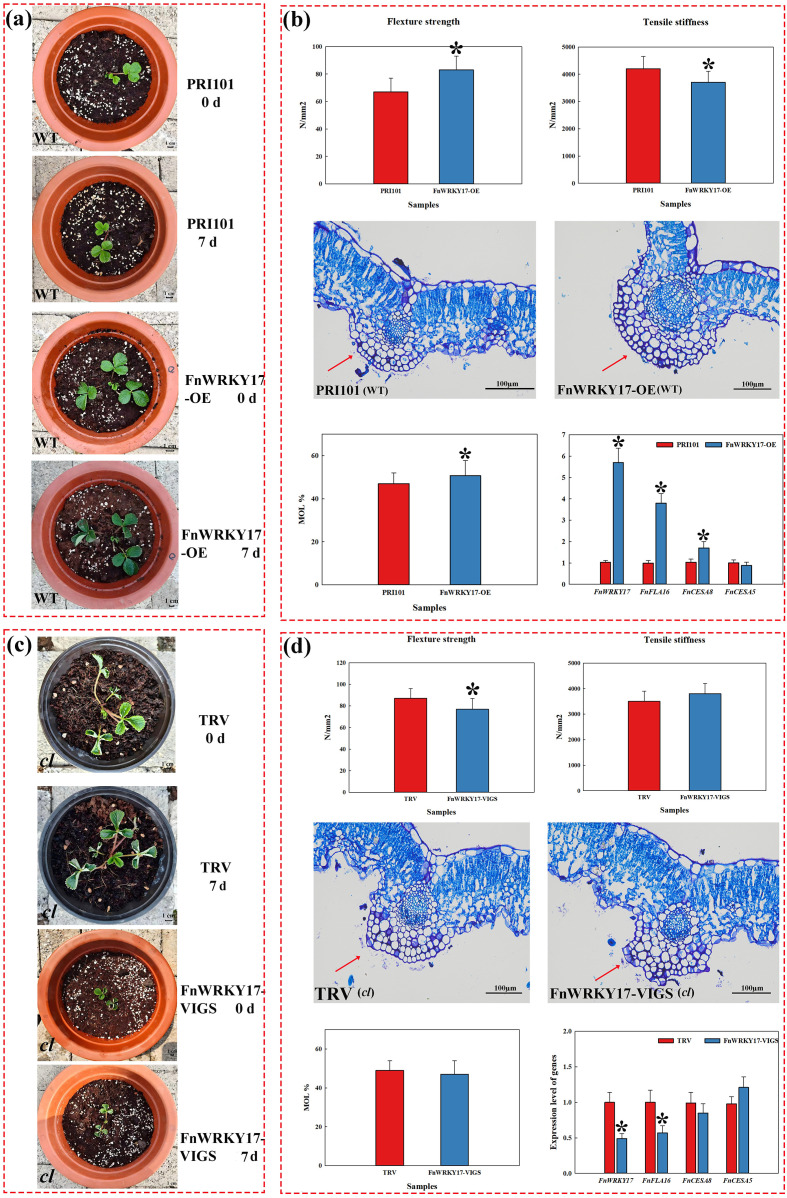
FnWRKY17 is correlated with leaf curling in the *cl* mutant of *F. nilgerrensis*. **(a)** Phenotype observation after 7 days of *Agrobacterium*-mediated transient transformation, Scale bar = 1 cm. Above: WT plants infiltrated with empty vector pRI101-empty (negative control); below: WT plants overexpressing *FnWRKY17* (FnWRKY17-OE). **(b)** Biomechanical and histological analyses of leaves from pRI101-empty and FnWRKY17-OE plants (n = 3 biological replicates per group, each replicate consisting of 3 technical measurements). Upper panel: flexure strength (left) and tensile stiffness (right). Middle panel: toluidine blue staining of transverse leaf sections showing cell wall morphology; arrows indicated the increased area of vascular bundles and the increased number of vascular bundle cells in FnWRKY17-OE transiently transformed plants. Scale bar = 100 μm. Lower panel left: cellulose content. Lower panel right: relative expression levels of cellulose synthase-related genes (*FnCESA5*, *FnCESA8 FnFLA16* and *FnWRKY17*) measured by qRT-PCR. **(c)** Phenotype observation at 7 days post-infiltration with TRV-based virus-induced gene silencing (VIGS), Scale bar = 1 cm. Above: *cl* plants infiltrated with empty vector control (TRV1-TRV2) (negative control); below: *cl* plants infiltrated with TRV1-TRV2-*FnWRKY17* (FnWRKY17-VIGS). **(d)** Biomechanical and histological analyses of leaves from TRV1-TRV2 and FnWRKY17-VIGS plants. Upper panel: flexure strength (left) and tensile stiffness (right). Middle panel: toluidine blue staining of transverse leaf sections showing cell wall morphology; arrows indicated the decreased area of vascular bundles and the decreased number of vascular bundle cells in VIGS transiently transformed plants. Scale bar = 100 μm. Lower panel left: cellulose content. Lower panel right: relative expression levels of cellulose synthase-related genes (*FnCESA5*, *FnCESA8 FnFLA16* and *FnWRKY17*) measured by qRT-PCR. Data were presented as mean ± SD. ‘*’ indicated significant differences among treatments according to Student’s t test (*P* < 0.05).

*FnWRKY17-*overexpressing and silencing constructs were transiently introduced into WT and *cl* leaves via *Agrobacterium*-mediated transformation to further clarify the involvement of *FnWRKY17* in leaf curling in the *cl* mutant. Phenotypes and related parameters were assessed 7 days after infiltration. *FnWRKY17-*OE leaves exhibited more pronounced curling than CK (empty PRI101) (**Figure 7a**). The flexural strength increased in FnWRKY17-OE leaves relative to CK, whereas the tensile stiffness declined (**Figure 7b**). Polysaccharide analysis indicated elevated cellulose content in FnWRKY17-OE leaves (**Figure 7b**). qRT–PCR revealed a significantly higher abundance of *FnFLA16* transcript in FnWRKY17-OE leaves (**Figure 7b**). Among the cellulose synthase genes, *FnCESA8* expression was markedly elevated, whereas *FnCESA5* expression was reduced (**Figure 7b**). Toluidine blue staining further showed increased vascular bundle area and cell number in FnWRKY17-OE transiently transformed plants compared with CK (**Figure 7b**).

*FnWRKY17* was also transiently silenced in the *cl* mutant using the TRV1-TRV2-FnWRKY17 vector, with TRV1-TRV2 serving as the control (CK). After 7 days of injections with FnWRKY7-VIGS, the curling phenomenon had somewhat alleviation compared with CK transiently transformed plants (**Figure 7c**), and the toluidine blue staining indicated the vascular bundle area and cell number in FnWRKY17-VIGS transiently transformed plants decreased compared to CK (**Figure 7d**). And the biomechanical properties displayed trends opposite to those observed in the FnWRKY17-OE transiently transformed plants (**Figure 7d**). The FnWRKY17-VIGS transiently transformed plants exhibited a significant reduction in flexural strength and *FnWRKY17/FnFLA16* expression levels compared with the CK. Cellulose content was reduced relative to CK (**Figure 7d**) and gene expression patterns showed inverse trends compared with FnWRKY17-OE (**Figure 7d**), although no significant differences were detected. Collectively, these findings demonstrate that FnWRKY17 binds to the *FnFLA16* promoter and modulates leaf biological properties by regulating cellulose content in the cell wall, thereby contributing to leaf curling in *F. nilgerrensis*.

## Discussion

### Role of *FnFLA16* expression in leaf curling regulation

Fasciclin-like arabinogalactan proteins (FLAs) constitute a distinct subgroup within the AGP superfamily and are characterized by the presence of one or two fasciclin domains. In *Arabidopsis thaliana*, FLA genes are classified into four phylogenetic subgroups (A–D), primarily distinguished by the number of FAS1 domains and the presence or absence of a GPI anchor signal. A large number of studies have shown that the FLA family genes regulate plant growth and development and resistance. In Arabidopsis, FLA4 glycosylation is required for normal root growth (Xue et al., 2017). *FLA3* RNAi (RNA interference) and overexpression indicated its role in pollen development (Li et al., 2010); *fla1* mutants showed impaired shoot regeneration *in vitro* (Johnson et al., 2011); *fla9* mutants exhibit increased seed abortion under drought (Cagnola et al., 2018). *FLA16* was required for stem development in *Arabidopsis* (Liu et al., 2020). FLAs are thought to modulate the organization of cell wall polysaccharides (e.g., cellulose and pectins), thereby altering wall properties and plant growth (MacMillan et al., 2010; Griffiths et al., 2016). FLAs may also interact with receptor-like kinases (RLKs) to activate signaling pathways that maintain cell wall integrity (Basu et al., 2016). Genetic interaction studies place *fla4*/*sos5* and the *FEI1/FEI2 RLK* mutants in a linear pathway controlling root development (Basu et al., 2016; Griffiths et al., 2016).

**Figure 8:**
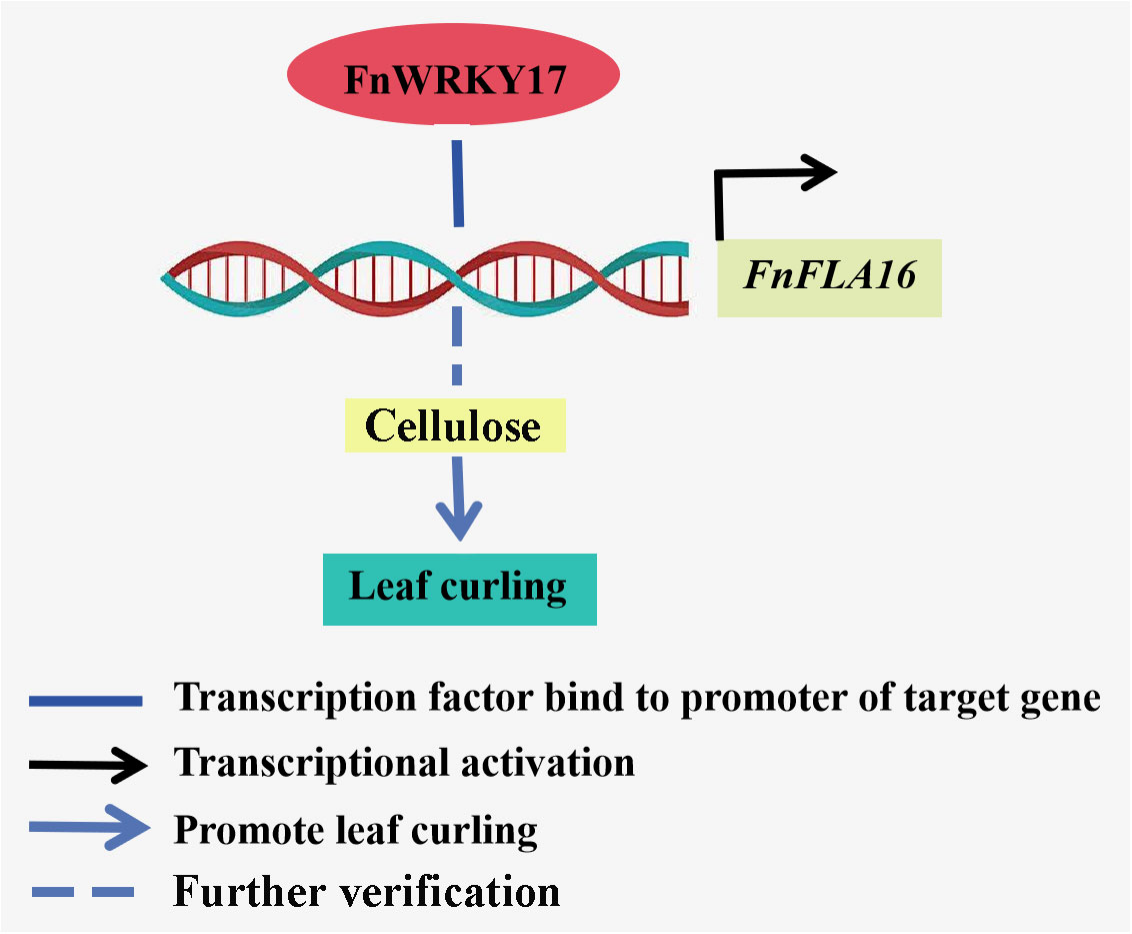
The putative working model of FnWRKY17-FnFLA16 modulated leaf curling in *cl* mutant of *F. nilgerrensis*. Proposed model for FnWRKY17-FnFLA16 regulation leaf curling through cellulose metabolism of *cl* mutant in *F. nilgerrensis.*.

Group B FLAs typically contain two FAS1 domains and lack a GPI anchor, localizing to the plasma membrane-cell wall interface, where they influence secondary wall formation and biomechanical properties through modulation of cell wall polymer deposition (MacMillan et al., 2010, 2015; Wang et al., 2015). In *Populus trichocarpa* (Wang et al., 2015), as well as in cotton fiber development (*Gossypium hirsutum*) (MacMillan et al., 2017) and tension wood formation in *Populus tremula* (Bygdell et al., 2017), group B FLA members showed preferential and elevated expression in these tissues, where they contribute to the regulation of tissue-specific biological properties. For instance, *FLA16* in *A. thaliana* plays a key role in maintaining stem mechanical strength by regulating cellulose deposition in the secondary cell wall (Liu et al., 2020). In this study, FnFLA16, a group B FLA in *F. nilgerrensis*, was identified as a novel regulator of leaf biomechanics and cellulose accumulation in the *cl* mutant. Subcellular localization analysis showed that *FnFLA16* was associated with the cell wall and the plasma membrane – cell wall interface in cells with secondary wall development (**Figure 3e**). *FnFLA16* overexpression resulted in reduced upper epidermal cell size, increased upper epidermal cell number and vascular bundle area, and pronounced alterations in biomechanical characteristics, including flexural strength and tensile stiffness, compared with CK. These findings differ from those of previous reports in which *FLA16* expression was mainly associated with stem secondary wall tissues and stem biomechanics regulation (MacMillan et al., 2015). This suggests a previously uncharacterized function of *FnFLA16* in modulating leaf-specific structural and mechanical traits. Furthermore, both FnFLA16-OE and Virus-induced gene silencing (VIGS) altered the cellulose content relative to CK. The expression levels of key cellulose biosynthetic genes (*FnCESA3*, *FnCESA5*, *FnCESA7*, and *FnCESA8*) were significantly affected. A large number of studies have shown that *FLAs* play an important role in altering the biological characteristics of tissues by regulating cell wall components, including *Arabidopsis* (Liu et al., 2020), *Oryza sativa* (Jiang et al., 2022a), and *Populus* (Wang et al., 2015). Regulation of *OreFLA11* expression via genetic engineering approaches can alter the flexural strength and stiffness of tissues by altering the cell wall component of the SCW in *Ostrya rehderiana* (Niu et al., 2024). Therefore, it is proposed that FnFLAs regulate leaf biological characteristics in *F. nilgerrensis* leaves by modulating cell wall cellulose content (**Figure 8**). However, the underlying regulatory mechanism requires further investigation.

### Role of cellulose content in leaf curling regulation

Cellulose is the major component of secondary walls, and a large number of studies have shown the leaves curling lead to significant changes in the cellulose content of the cell walls. For example, *OsCD1* and *OsCSLD4* encoded putative member of the cellulose synthase-like D sub-family and were essential for rice curled leaf morphology (Luan et al., 2011; Qiao et al., 2023). In addition, cellulose a key factor influencing mechanical properties (Turner and Somerville, 1997; Wu et al., 2025). For example, the GPI-anchored COBRA-like proteins can directly bind cellulose and regulate microfibril crystallinity in *Arabidopsis* (Roudier et al., 2002), rice (Liu et al., 2013), and maize (Sindhu et al., 2007), and mutants have reduced mechanical strength. Therefore, there is a strong correlation among leaf curling, mechanical and mechanical properties, and cellulose content. Here, altered cellulose levels were observed in the *cl* mutant. The reduced tensile stiffness in *cl* leaves was consistent with the increased cellulose content and flexural strength. A similar trend was observed in the FnFLA16-OE transiently transformed plants, whereas the opposite pattern was detected in the FnFLA16-VIGS transiently transformed plants. These findings indicate that alterations in cell wall matrix components (e.g., hemicellulose) (Kohler and Spatz, 2002) and cell wall structural organization (cellulose) may be responsible for the changes in biological properties (Cave and Walker, 1994; Keckes et al., 2003), with FLAs playing a key regulatory role in this process (**Figure 8**) (Liu et al., 2020). Transcript levels of *FnCESA3*, *FnCESA5*, *FnCESA7*, and *FnCESA8* were altered in *FnFLA16* transiently transformed plants, with *FnCESA8* showing consistent and significant changes in both FLA16-OE and FLA16-VIGS transiently transformed plants. Previous studies have reported that *CESA8* mutations lead to the irregular xylem (*irx1*) phenotype, which is characterized by xylem cell atrophy (Taylor et al., 2000). Compared to WT, this phenotype exhibits up to 40% reduction in stem cellulose, accompanied by altered mechanical properties (Turner and Somerville, 1997; Taylor et al., 2000). Therefore, *FnCESAs*, particularly *FnCESA8*, may be the main regulators underlying changes in the leaf biological characteristics of strawberry and cellulose variations in the cell wall content mediated by FnFLA16. Furthermore, FLAs are capable of mediating protein-protein, protein-carbohydrate, and carbohydrate-carbohydrate interactions, suggesting multifunctional roles in signaling pathways and cell wall structure (Wolf et al., 2012). Therefore, while the expression level of *FLA16* likely affect the cellulose content, the precise mechanism by which FLAs regulates the cellulose content obscure. Existing studies indicates that FLAs may be present in all layers of the xylem secondary cell wall (SCW), thereby may play a significant role in promoting adhesion between the major structural components of the SCW and affect the content of cellulose (MacMillan et al., 2015). Another possibility is that FLA16 play a role in signal transduction between the intracellular region and the extracellular matrix, surrounding cells, and the external environment; alterations in the orientation of microtubule arrays via interactions with FLA16 in lipid rafts might affect cellulose deposition (MacMillan et al., 2015). It is hypothesized that FnFLA16 influences cellulose metabolism at multiple regulatory levels, potentially by altering cell wall polysaccharide composition or functioning as a signaling component. However, the mechanism by which altered FnFLA16 abundance affects *FnCESAs* transcription remains unclear. Further investigation is needed to elucidate how *FnFLA16*-mediated cellulose content modulation contributes to leaf curling in *F. nilgerrensis*.

### FnWRKY17 regulates *FnFLA16* expression to promote leaf curling

WRKY transcription factors are one of the largest families of plant transcriptional regulators and modulate diverse developmental and stress-responsive pathways by binding to promoter regions of target genes (Li et al., 2015; Miyamoto et al., 2020). MdWRKY17, GhWRKY21, MsWRKY11, and PoWRKY17 directly regulate drought resistance in *Malus domestica*, *Gossypium hirsutum*, *Medicago sativa*, and *Paeonia ostii*, respectively (Wang et al., 2020; Shan et al., 2021; Wen et al., 2021; Luan et al., 2023). Here, Y1H, LUC, and GUS assays demonstrated that FnWRKY17 activates *FnFLA16* transcription. FnWRKY17 overexpression resulted in elevated *FnFLA16* expression, accompanied by phenotypic, biomechanical, cellulose content, and cellulose metabolism gene expression patterns similar to those observed in FnFLA16-OE transiently transformed plants. In contrast, opposite trends were observed in the FnWRKY17-silenced transiently transformed plants. However, the phenotypic effects of FnWRKY17 silencing was not significant. The possible reasons for the insignificant effect is that FnWRKY17 may not be the only regulatory factor for the target gene *FnFLA16*. There may be other TFs with redundant functions, or the target gene is regulated by multiple transcription factors together. When the FnWRKY17 was silenced, other factors may partially compensate, resulting in only a slight decrease in expression without being significant. Additionally, the silencing effect of the FnWRKY17 may not be ideal. If the silencing was not thorough, the residual transcription factor activity may remain at a level close to the normal expression level, thus causing only a slight decrease in the expression of the target gene *FnFLA16*, and the change in cellulose content has not accumulated to the extent that it can change the phenotypic morphology.

Moreove, transcript levels of *FnWRKY17* and *FnFLA16* were found to be elevated in the *cl* mutant. Given that their genomic sequences remain intact, the EMS-induced mutation most likely affects an upstream transcription factor or a signaling component that positively regulates *FnWRKY17* expression. Although the *cl* mutation does not reside within *FnWRKY17* or *FnFLA16*, overexpression of either *FnWRKY17* or *FnFLA16* in the wild-type background induced the leaf-curling phenotype and led to increased cellulose content (revised **Figures 4**, **7**). The mechanistic relationship between the FnWRKY17–FnFLA16 complex was further validated using multiple assays, including Y1H, LUC, and GUS reporter analyses. Taken together, our current data support a model where EMS mutagenesis was likely to enhance the expression level of *FnWRKY17*, thereby promoting the FnWRKY17-FnFLA16 transcription complex, facilitating the cellulose synthase gene, and leading to an increase in cellulose content and leaf curling. These findings indicate that FnWRKY17 acts upstream of *FnFLA16* and participates in FnFLA16-mediated regulation of leaf curling in *F. nilgerrensis* (**Figure 8**), suggesting that the FnWRKY17–FnFLA16–cellulose module may underpin the curled phenotype. Currently, whole-genome sequencing is being used to identify the key mutation sites in genes responsible for the phenotypic changes.

## Conclusions

This study clarifies the working model of leaf curling in the *cl* mutant of *F. nilgerrensis*. Alterations in cell wall structure and cellulose composition, together with changes in biological characteristics, were associated with the leaf curling phenotype. RNA sequencing analysis identified differential *FLA* gene expression between *cl* and WT plants, and functional assays confirmed that *FnFLA16* contributes to leaf curling by modulating cell wall cellulose composition. Furthermore, Y1H, LUC, and GUS assays demonstrated that FnWRKY17 activates *FnFLA16* transcription, whereas overexpression and silencing experiments verified the involvement of *FnWRKY17* in *FnFLA16*-mediated regulation of leaf curling. Collectively, these findings support a working model explaining leaf curling in the *F. nilgerrensis cl* mutant. The results of this study provide mechanistic insights into the regulatory pathways underlying leaf curling, establish a foundation for the functional analysis of key associated genes, and offer potential guidance for the breeding of *F. nilgerrensis* cultivars with enhanced drought resistance.

## Data Availability

The datasets presented in this study can be found in online repositories. The names of the repository/repositories and accession number(s) can be found in the article/**Supplementary Material**.

## Supplementary material

Supplementary Table 1 Primers used in the experiment.

Supplementary Table 2 Summary of the sequencing and mapping of 6 samples.

Supplementary Table 3 Key DEGs in *cl* mutant.

Supplementary Table 4 TFs-DEGs relative analysis.

Supplementary Figure 1 Instantaneous infection efficiency.
