# Supplementary figures and images for: A FnWRKY17–FnFLA16 regulatory module controls leaf curling in Fragaria nilgerrensis
Source: Front Plant Sci. 2026 Jun 23;17:1837692. doi: 10.3389/fpls.2026.1837692 (PMC13337782; doi:10.3389/fpls.2026.1837692)

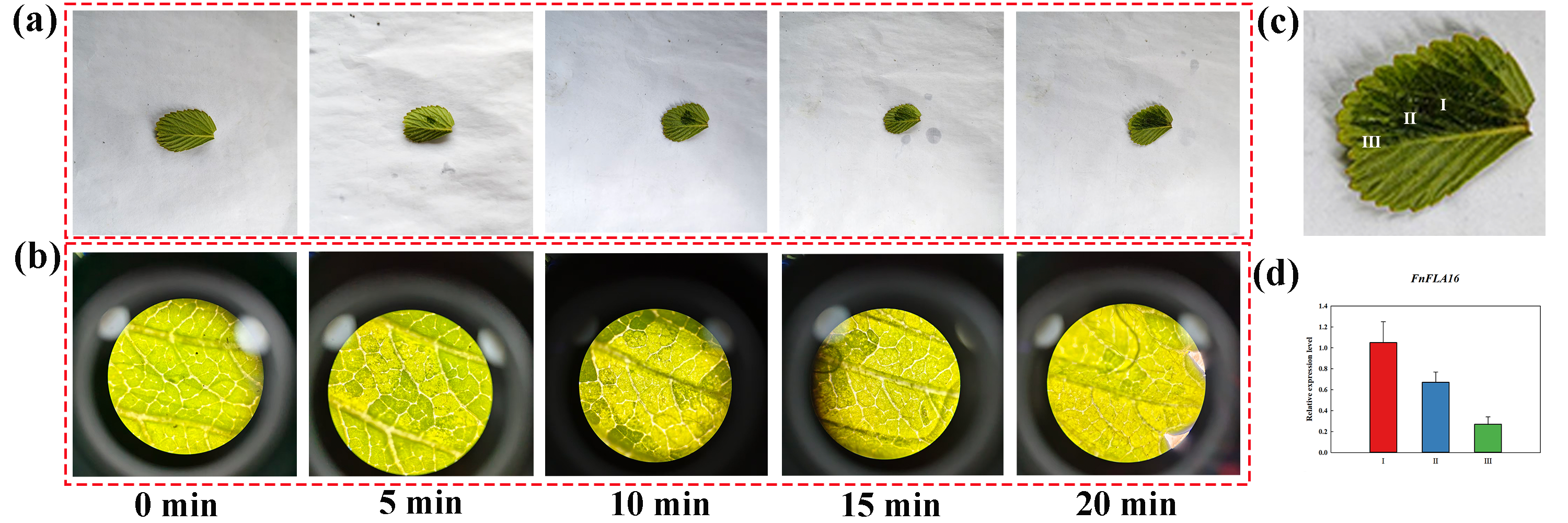

Supplement: Supplementary Figure 1 — Instantaneous infection efficiency. [file Image1.tif]
